# A polysaccharide from *Lentinus edodes* inhibits human colon cancer cell proliferation and suppresses tumor growth in athymic nude mice

**DOI:** 10.18632/oncotarget.13481

**Published:** 2016-11-21

**Authors:** Jinglin Wang, Weiyong Li, Xiao Huang, Ying Liu, Qiang Li, Ziming Zheng, Kaiping Wang

**Affiliations:** ^1^ Union Hospital of Huazhong University of Science and Technology, Department of Pharmacy, 430030, Wuhan, China; ^2^ Hubei Key Laboratory of Nature Medicinal Chemistry and Resource Evaluation, Tongji Medical College of Pharmacy, Huazhong University of Science and Technology, 430030, Wuhan, China; ^3^ Renmin Hospital of Wuhan University, Department of Pharmacy, 430060, Wuhan, China

**Keywords:** apoptosis, colon cancer, lentinan, nude mice, ROS

## Abstract

The antitumor effect of Lentinan is thought rely on the activation of immune responses; however, little is known about whether Lentinan also directly attacks cancer cells. We therefore investigated the direct antitumor activity of SLNT (a water-extracted polysaccharide from Lentinus edodes) and its probable mechanism. We showed that SLNT significantly inhibited proliferation of HT-29 colon cancer cells and suppressed tumor growth in nude mice. Annxein V-FITC/PI, DAPI, AO/EB and H&E staining assays all showed that SLNT induced cell apoptosis both *in vitro* and *in vivo*. SLNT induced apoptosis by activating Caspase-3 via both intrinsic and extrinsic pathways, which presented as the activation of Caspases-9 and -8, upregulation of cytochrome c and the Bax/Bcl-2 ratio, downregulation of NF-κB, and overproduction of ROS and TNF-α *in vitro* and *in vivo*. Pretreatment with the caspase-3 inhibitor Ac-DEVD-CHO or antioxidant NAC blocked SLNT-induced apoptosis. These findings suggest that SLNT exerts direct antitumor effects by inducing cell apoptosis via ROS-mediated intrinsic and TNF-α-mediated extrinsic pathways. SLNT may thus represent a useful candidate for colon cancer prevention and treatment.

## INTRODUCTION

Colon cancer is the second leading cause of cancer-related deaths worldwide, accounting for over 1 million new cases and about half a million deaths per year [1, 2]. Surgery is the primary treatment for colon cancer. For most patients with metastasis, however, systemic chemotherapy is needed to relieve symptoms and prolong life. Standard first-line chemotherapeutic regimens for colon cancer involved a combination of infusional 5-fluorouracil (5-FU), leucovorin and oxaliplatin or 5-FU, irinotecan and bevacizumab [3, 4]. However, theside effects associated with these chemotherapeutic strategies, which include diarrhea, nausea and vomiting, acute myocardial infarction and cerebrovascular accident, greatly decrease the patients' quality of life and can be fatal at times [5]. There is thus a great need to screen for natural products exhibiting antitumor activities with low toxicity and high efficacy.

*Lentinus edodes* (called Xianggu and Shiitake in China and Japan respectively) is a popular edible mushroom in East Asia and is frequently used in food and in folk medicine. As a foodstuff, it is appetizing and nourishing and has been cultivated for thousands of years. As a medicinal agent, *L. edodes* exhibits antifungal/antibacterial, antiviral, antioxidant, immunomodulatory and antitumor activities [6]. Among the various bioactive molecules responsible for these activities, polysaccharides are the best known and most potent mushroom-derived substances. Lentinan, a β-1, 3-glucan polysaccharide isolated from *L. edodes*, has been used clinically in Japan since the early 1980s because of its immunomodulatory and antitumor effects [7–9]. Earlier findings suggest Lentinan exerts its antitumor effects by activating immune responses in the host rather than directly by attacking cancer cells. The antitumor activity of Lentinan was thought to require an intact T-cell component and was mediated through a thymus-dependent immune mechanism [10]. However, recent studies have shown that Lentinan directly inhibits proliferation of liver cancer cells (HepG2) [11], murine skin carcinoma cells (CH72) [12], and breast cancer cells (MCF-7) [13]. However, little is known about the mechanism underlying the direct antitumor activity of Lentinan.

In previous studies from our laboratory, water-extracted and alkali-extracted polysaccharides were isolated from the fruit bodies of *L. edodes,* and their chemical structures and conformations were determined [11, 14]. Studies showed that the antitumor activity of Lentinan in H22- and S180-bearing Balb/c mice reflected immunomodulatory activity and induction of apoptosis [11, 15]. Thus, in the current study, we explored the direct antitumor activity of SLNT (a water-extracted polysaccharide from *L.edodes*) on HT-29 human colon cancer cells and HT-29 tumor-bearing athymic nude mice to assess its direct antitumor effects in the absence of an intact T-cell component and shed new light on its mechanism of action.

## RESULTS

### SLNT inhibited the proliferation of HT-29 cells and induced apoptosis

**Figure 1:**
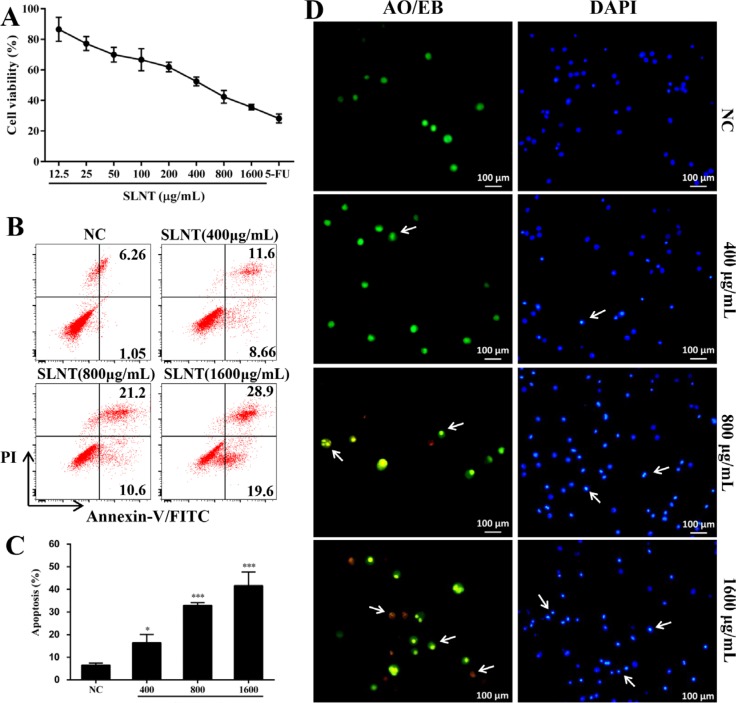
SLNT inhibited HT-29 cell proliferation and induced apoptosis The cell viability was evaluated by MTT assay (**A**). Annexin V-FITC/PI staining was used to analyze apoptosis; representative results (**B**) and summative data (**C**) from three independent experiments were shown. Cells treated with 400, 800 and 1600 μg/mL of SLNT or without for 48 h were stained with AO/EB (green/orange) or DAPI (blue) (**D**). Each bar represents means ± SD (*n* = 3). ^*^p < 0.05, ^***^p < 0.001 versus negative control (NC) group.

To investigate the effects of water-extracted Lentinan (SLNT) on the proliferation of HT-29 cells, MTT assay was performed. A concentration dependent inhibitory effect was observed when HT-29 cells were exposed to SLNT (0–1600 μg/mL) for 48 h (Figure 1A). SLNT (1600 μg/mL) exhibited a strongest direct antitumor activity on HT-29 cells with 35.57% of cell viability.

Owing to apoptosis is considered as the main cell death mechanism, we next tested apoptosis by flow cytometry. In Figure 1B and 1C, SLNT significantly induced HT-29 cell apoptosis in a dose-dependent manner, with 20.26%, 31.80% and 48.50% of apoptotic rate, respectively, compared with the negative control (NC, 7.31%). To further confirm it, AO/EB and DAPI staining were performed. In AO/EB staining, the images (Figure 1D) revealed that more apoptotic cells (condensed green or orange) and less normal cells (uniform green) existed in SLNT group than NC. In DAPI staining (a nuclear stain), NC cells displayed homogenous fluorescence with no evidence of fragmentation or chromatin condensation (Figure 1D) SLNT-treated cells had prominent DNA condensation. Altogether, these results showed that SLNT induced HT-29 cell apoptosis thereby exerting its direct antitumor effects.

### SLNT suppressed HT-29 tumor growth in nude mice and induced apoptosis

**Figure 2:**
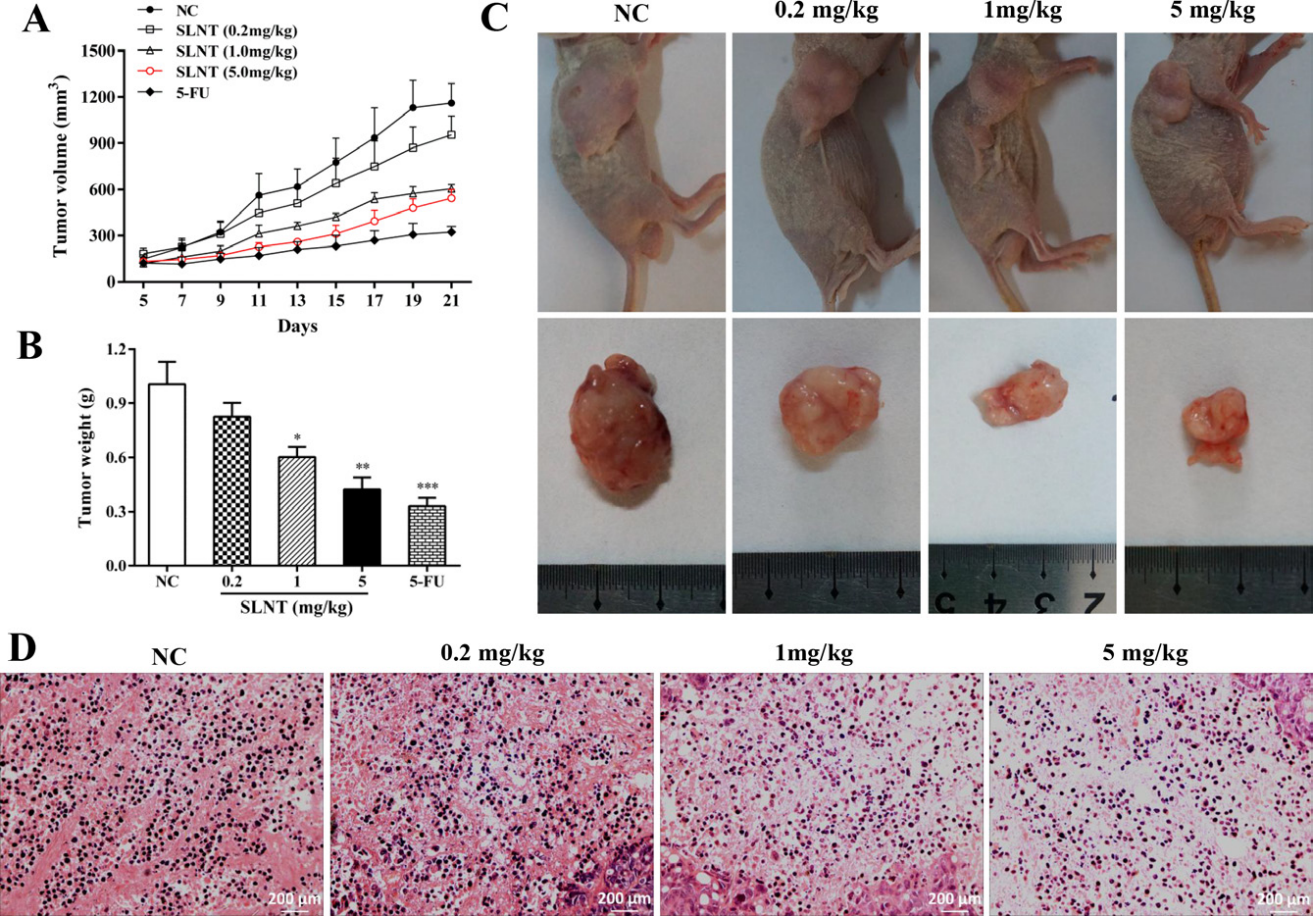
Inhibition and apoptotic induction effect of SLNT on HT-29 xenografts Tumor volume from day 5 to day 21 (**A**) and tumor weights at the end of treatments were measured (**B**) Columns, mean ± SD (*n* = 6); ^*^p < 0.05, ^**^p < 0.01, ^***^p < 0.001 versus NC group. Photographs of nude mice and tumors of each group were taken at end of experiment (**C**) Representative histopathological changes of apoptosis in tumors were analyzed by H&E staining (400×, **D**).

We next investigated the effect of SLNT *in vivo*. Tumor volumes (Figure 2A) and the final tumor weights (Figure 2B) of mice were shown. Surprisedly, tumors in SLNT-receiving mice were smaller than that of NC mice. And the inhibition rates of SLNT (0.2, 1 and 5 mg/kg) were 17.88%, 48.87% and 57.90%, respectively, while 5-FU was 67.23%, compared with the NC (Table 1). These suggested that tumor growth was efficiently retarded in SLNT-receiving mice compared with unreceiving mice. Additionally, the photographs of mice and tumors took at the end of treatment also clearly showed the arrest of tumor growth (Figure 2C). Nevertheless, no changes were observed in spleen index (Table 1).

**Table 1:** The final tumor weight, inhibition rate and spleen index of nude mice

	Final tumor weight (g)	Inhibition rate (%)	Spleen index (mg/g)
Negative control	1.01 ± 0.27	_	1.13 ± 0.09
SLNT (0.2 mg/kg)	0.83 ± 0.17	17.88	1.32 ± 0.28
SLNT (1.0 mg/kg)	0.60 ± 0.13^*^	48.87	1.19 ± 0.15
SLNT (5.0 mg/kg)	0.42 ± 0.15^**^	57.90	1.16 ± 0.12
5-FU (20 mg/kg)	0.33 ± 0.10^**^	67.23	1.22 ± 0.40

SLNT and 5-FU were administered intravenously every other day for 21 days, 24 h after subcutaneously injection of HT-29 cells. Mice were sacrificed 3 weeks after treatments. Inhibition rate was measured as described in Materials and methods. Spleen index was expressed as: spleen weight/body weight. All data are presented as means ± SD (*n* = 6).

* *p* < 0.05,

** *p* < 0.01 versus NC.

To further explore whether apoptosis was involved in prevention of tumor growth, we analyzed tumor tissues by H&E staining. Marked apoptotic morphologic changes such as karyorrhexis and irregular arrangement of karyomorphism were observed in SLNT group. Conversely, NC group kept good original grown status with completed, well-regulated and clearly visible karyomorphism (Figure 2D). This suggested that SLNT also induced apoptosis in HT-29 tumor xenografts.

### SLNT activated Caspase-3 in HT-29 cells and in tumor xenografts

**Figure 3:**
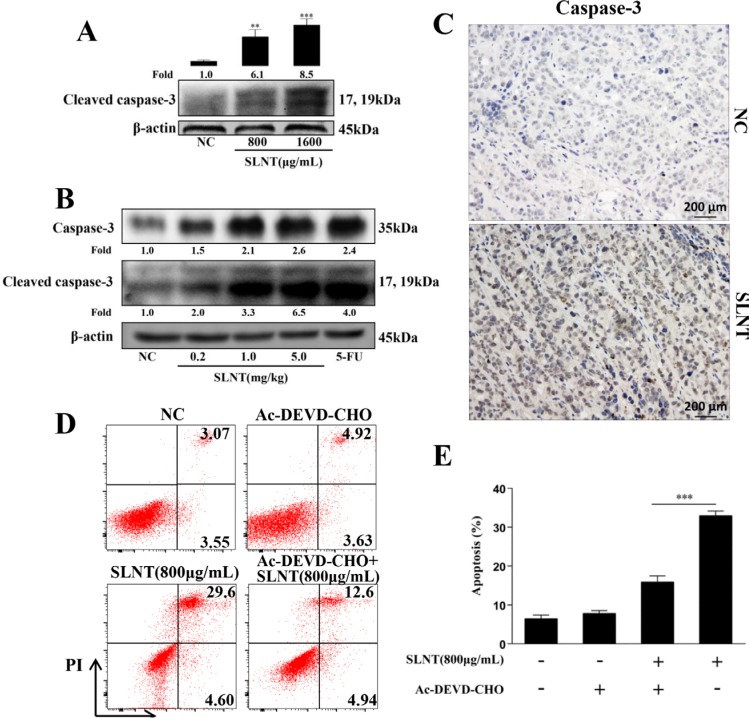
SLNT induced HT-29 cell apoptosis by activation of caspase-3 in cells and in tumors Activity of caspase-3 in cells (**A**) and in tumor xenografts (**B**) was measured by western blot. Immunohistochemistry analysis of caspase-3 in tumors of SLNT (5.0 mg/kg) and NC were shown (**C**, 400×). HT-29 cells were incubated with SLNT (800 μg/mL), a caspase-3 inhibitor Ac-DEVD-CHO (25 μM) or combination for 48 h, representative results (**D**) and summative data (**E**) were shown. Each bar represents means ± SD (*n* = 3). ^**^p < 0.01, ^***^p < 0.001 versus NC; ^###^*p* < 0.001 versus combination group.

To explore how SLNT induced HT-29 cell apoptosis, we examined the effect of SLNT on caspase-3, the key effector caspase which could induce apoptosis through cleavage of its substrates. Stimulation of HT-29 cells with SLNT caused a pronounced activation of caspase-3 (Figure 3A) while NC cells showed nearly no activation. Similar results were observed in tumor tissues measured by western blotting (Figure 3B) and immunohistochemistry (Figure 3C). These suggested that SLNT activated caspase-3 both *in vitro* and *in vivo*.

To ascertain the role of caspase-3 in SLNT-induced apoptosis, a caspase-3 inhibitor (Ac-DEVD-CHO) was used. HT-29 cells were pre-incubated with Ac-DEVD-CHO (25 μM) for 1 h before the addition of SLNT (800 μg/mL). In Figure 3D and 3E, the addition of Ac-DEVD-CHO significantly prevented SLNT-induced apoptosis (from 32.91 ± 1.21% decreased to 15.88 ± 1.58% while NC and Ac-DEVD-CHO group were 6.45 ± 0.96%, 7.77 ± 0.79%, respectively). However, SLNT+Ac-DEVD-CHO group (15.88 ± 1.58%) was still higher than Ac-DEVD-CHO group (7.77 ± 0.79%). These suggested that SLNT-induced apoptosis depended to a large degree on the activation of caspase-3.

### SLNT activated caspasese-9 and upregulated cytosolic Cytochrome c and the ratio of Bax/Bcl-2

**Figure 4:**
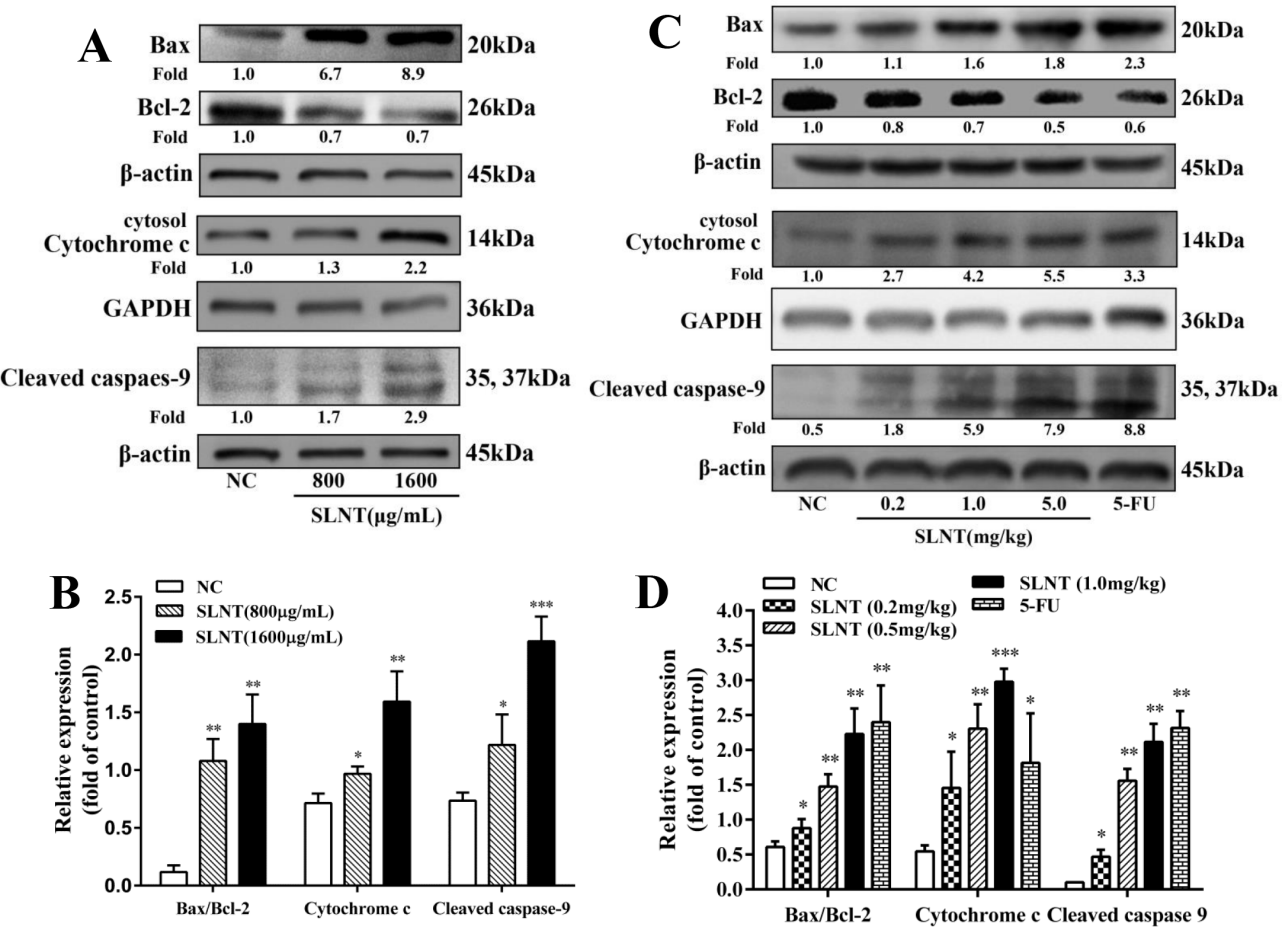
Effect of SLNT on intrinsic apoptotic-related proteins in cells and in tumors Expression of Bax, Bcl-2, cytosolic Cytochrome c and Cleaved caspase-9 in cells (**A, B**) and in tumor xenografts (**C, D**) were analyzed by western blot in three independent experiments. Each bar represents means ± SD (*n* = 3), ^*^p < 0.05, ^**^p < 0.01, ^***^p < 0.001 versus NC group.

We next examined the effect of SLNT on the intrinsic mitochondrial apoptosis-related proteins, cleaved caspase-9, cytochrome c, Bax and Bcl-2. As shown in Figure 4A and 4B, caspase-9 was activated, cytosolic cytochrome c and Bax increased while Bcl-2 decreased in SLNT-treated cells compared to NC. The ratio of Bax/Bcl-2 of SLNT-treated cells was about 3 fold higher than untreated cells. Consistent results were also observed in SLNT-treated HT-29 tumor xenografts (Figure 4C and 4D). These suggested that SLNT might induce HT-29 cell apoptosis via mitochondrial apoptotic pathway.

### SLNT induced the loss of mitochondrial membrane potential (MMP)

**Figure 5:**
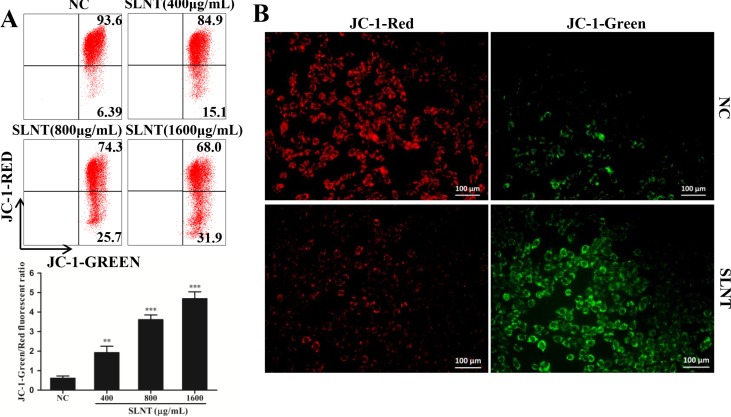
SLNT induced the loss of mitochondrial membrane potential (MMP) in cells The change of MMP was analyzed by flow cytometry (**A**) and fluorescence microscope (**B**, 400×). Representative images of NC and SLNT (800 μg/mL) taken by fluorescence microscope were shown.

Owing to the loss of MMP was a key event in the early apoptosis through mitochondrial apoptotic pathway, we detected the changes of MMP by JC-1 staining. Obviously, after incubated with SLNT, cells with green fluorescence significantly increased from 6.39% to 15.1%, 25.7% and 31.9% in order, and the ratio of green/red was remarkably higher than control (Figure 5A). The fluorescence pictures were shown (Figure 5B), which further confirmed the loss of ΔΨ_m_. These results suggested that SLNT indeed activated mitochondrial apoptotic pathway.

### SLNT increased the generation of ROS in HT-29 cells and in nude mice

**Figure 6:**
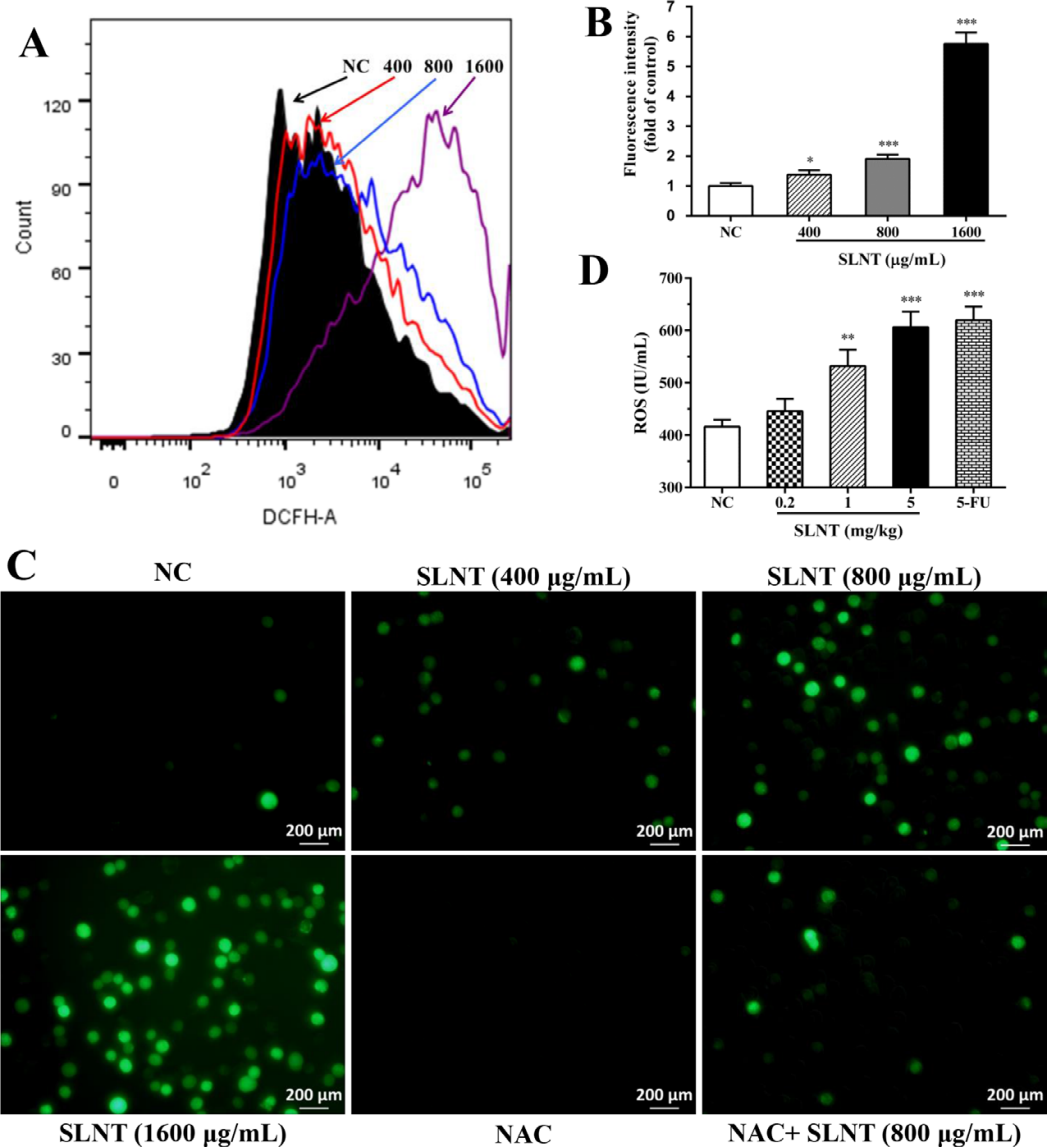
SLNT increased the generation of ROS in cells and in nude mice Cellular ROS was measured by DCFH-DA using flow cytometry: a mixture image of NC (black), 400 μg/mL (red), 800 μg/mL (blue) and 1600 μg/mL (purple) of SLNT was shown (**A**), and fluorescence intensity were summarized (**B**); representative images (**C**, 400×) were taken by fluorescence microscope. The level of ROS in serum of nude mice was detected by ELISA assay (**D**). Each bar represents means ± SD (*n* = 3–6), ^*^p < 0.05, ^**^p < 0.01, ^***^p < 0.001 versus NC group.

As ROS generated during oxidative stress is known to activate the intrinsic apoptosis, we next determined the effect of SLNT on generation of ROS. Compared to NC cells, SLNT significantly enhanced cellular ROS, particularly in a dose of 1600 μg/mL (Figure 6A and 6B). The photographs taken by fluorescence microscope also clearly showed increased ROS after stimulating with SLNT (Figure 6C). Additionally, significantly enhancement of ROS in serum of SLNT (1.0 and 5.0 mg/kg)-receiving mice were observed (Figure 6D).

**Figure 7:**
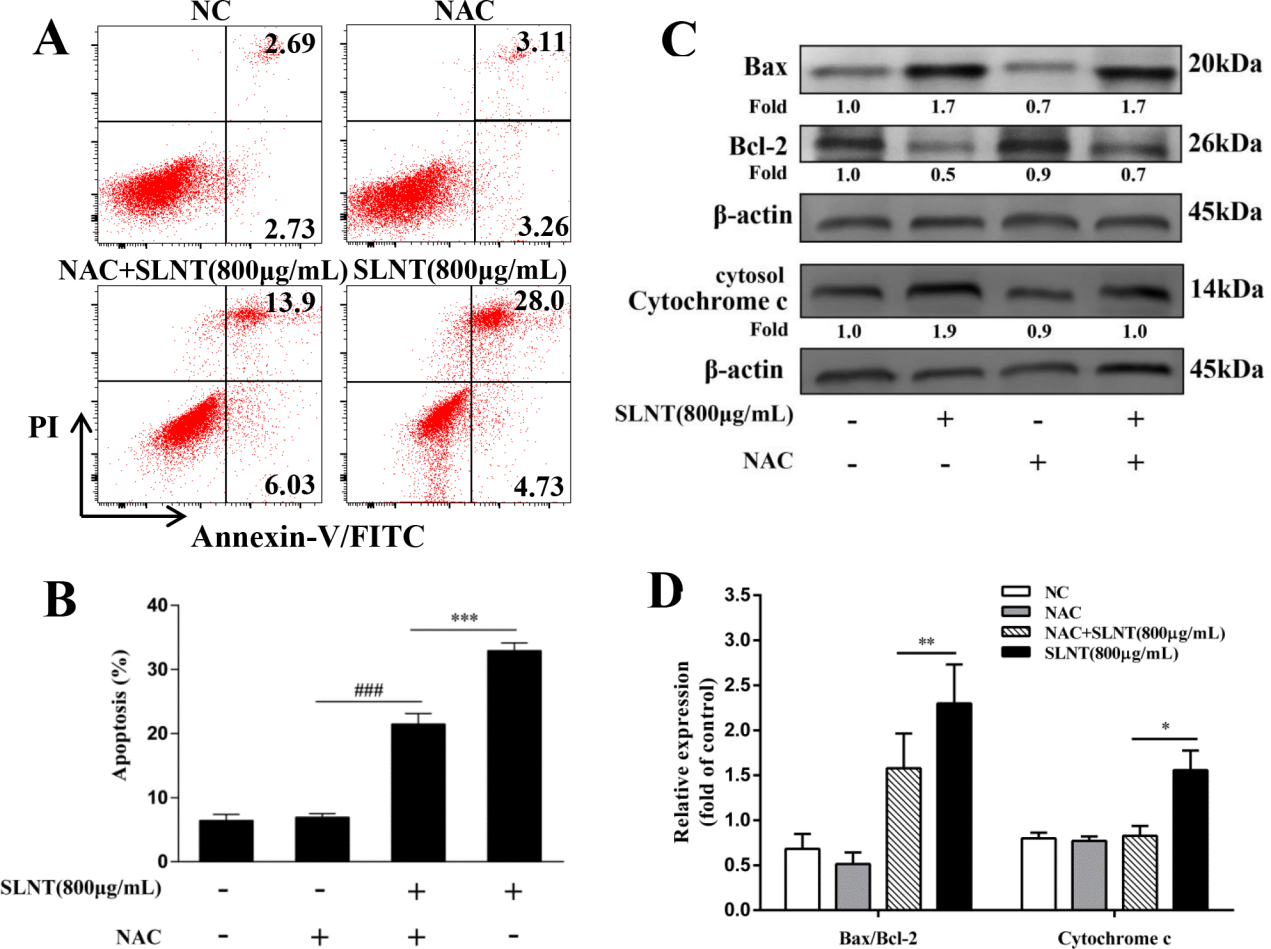
ROS was responsible for the SLNT-induced intrinsic apoptosis HT-29 cells were incubated with SLNT (800 μg/mL), an antioxidant NAC (5 μM), or combination for 48 h. Annexin V-FITC/PI staining was used to analyze cell apoptosis (**A, B**), ^***^p < 0.001 versus combination group, ^###^*p* < 0.001 versus NAC group. The expression of Bax, Bcl-2 and cytosol Cytochrome c were measured by western blot (**C**) and summarized (**D**). Each bar represents means ± SD (*n* = 3), ^*^p < 0.05, ^**^p < 0.01 versus combination group.

To evaluate whether the upregulation of ROS activated intrinsic apoptosis, an antioxidant NAC was used. HT-29 cells were pre-incubated with NAC (5 μM) for 1h before SLNT (800 μg/mL) was added. As shown in Figure 7A and 7B, the apoptotic rate of cells treated with SLNT (800 μg/mL) significantly decreased from 32.91 ± 1.21% to 21.49 ± 1.62% after pre-incubation of NAC, compared with NAC group (6.94 ± 0.57%). Meanwhile, SLNT-induced upregulation of cytosolic cytochrome c and Bax/Bcl-2 were blocked by NAC (Figure 7C and 7D). These results indicated that ROS might act as upstream for activating intrinsic pathway. However, interestingly, there were still about 20% apoptotic cells existed when ROS was largely cleaned out. This suggested that SLNT induced apoptosis maybe not only through intrinsic pathway, but also extrinsic pathway.

### SLNT activated Caspase-8 and prevented NF-κB activation and increased TNF-α levels *in vitro* and *in vivo*

**Figure 8:**
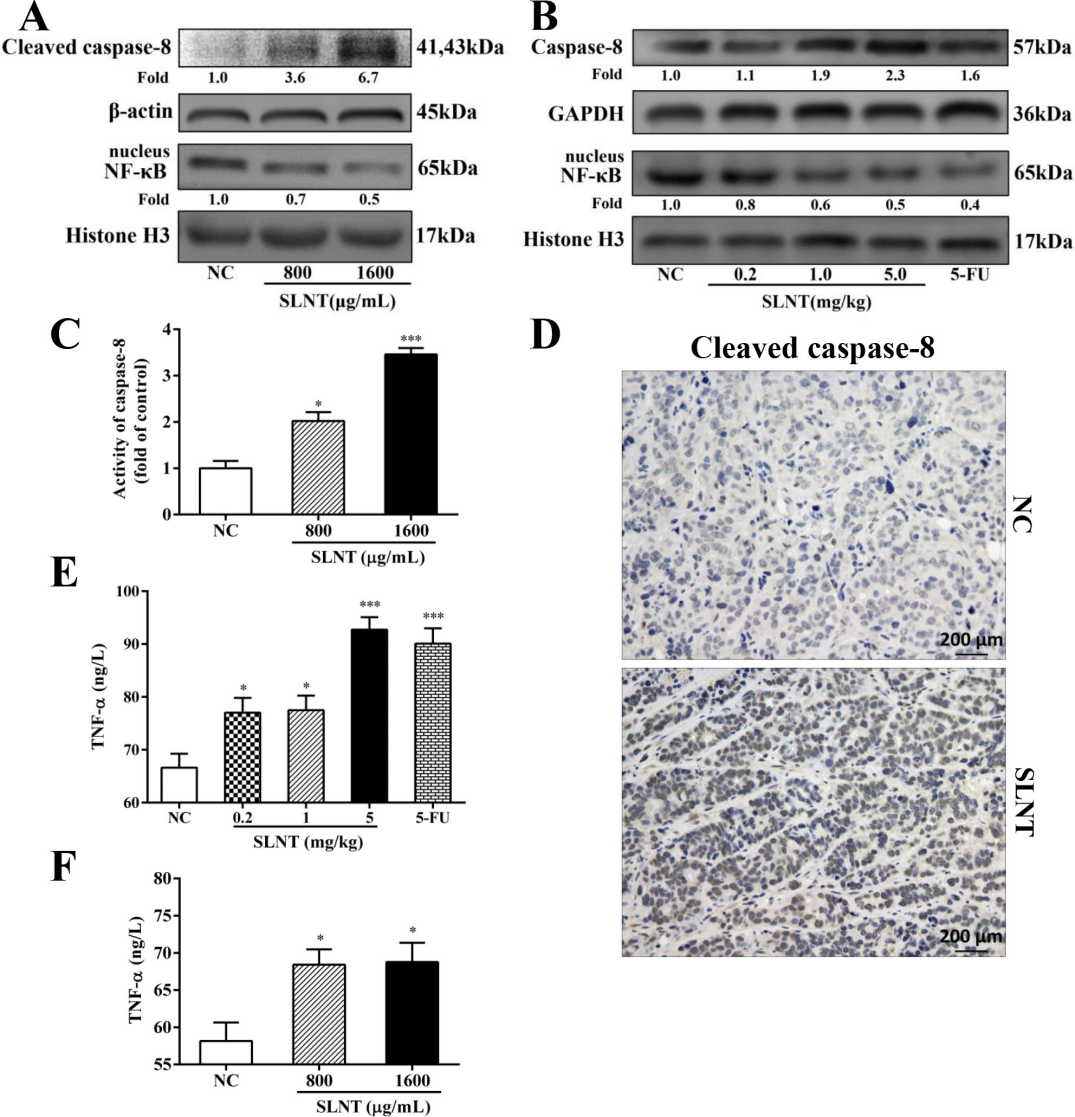
SLNT activated caspase-8, decreased nuclear NF-κB and increased TNF-α *in vitro* and *in vivo* Expression of cleaved caspase-8 and nuclear NF-κB in cells (**A**) and in tumors (**B**) were analyzed by western blot. Activity of caspase-8 in cells was further measured by Caspase-8 activity kit (**C**). Immunohistochemistry analysis of cleaved caspase-8 in NC and in SLNT (5.0 mg/kg) tumors was shown (**D**). The level of TNF-α in serum of nude mice (**E**) and in cell culture medium (**F**) was measured by ELISA assay. Each bar represents means ± SD, *n* = 3–6, ^*^p < 0.05, ^***^p < 0.001 versus NC group.

**Figure 9:**
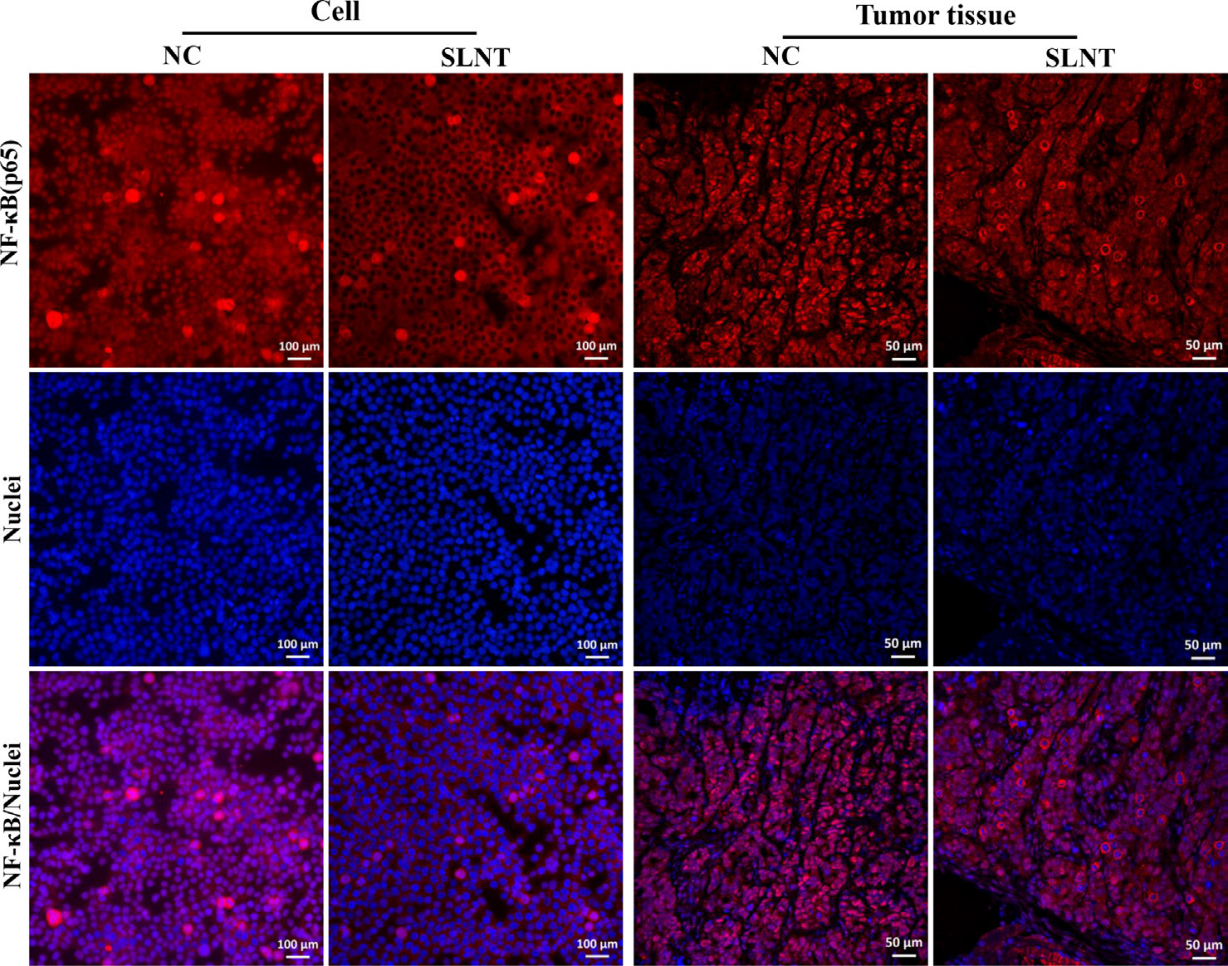
SLNT inhibited NF-κB p65 nuclear translocation both *in vitro* and *in vivo*

To ascertain whether extrinsic pathway was involved in SLNT-induced apoptosis, we analyzed activation of the key initiator caspase-8. Western blot showed that Caspase-8 was activated both *in vitro* (Figure 8A) and *in vivo* (Figure 8B). Additionally, Caspase-8 activity in cells and in tumors were analyzed by Caspase-8 colorimetric activity kit (Figure 8C) and immunohistochemistry (Figure 8D), respectively. The results showed that SLNT activated caspase-8 both *in vitro* and *in vivo*, suggesting SLNT might also activate caspase-8-mediated extrinsic apoptotic pathway. At the same time, we evaluated the nuclear translocation of NF-κB p65 using western blot and immunofluorescence. Western blot (Figure 8A and 8B) and immunofluorescence (Figure 9) revealed that SLNT induced an obvious decrease of NF-κB p65 nuclear translocation compared with NC both in cells and in tumors. Additionally, ELISA assay showed that TNF-α in serum (one of cytokines could activate extrinsic pathway) was enhanced after treated with SLNT (Figure 8E). We next measured TNF-α levels in cell culture medium after SLNT treatment. Results indicated that TNF-α was significantly increased after stimulated by SLNT, which were consistent with the outcomes *in vivo* (Figure 8F).

## DISCUSSION

Up to now, it was generally assumed that Lentinan mainly exerted its anti-tumor effects by enhancing immune responses, relying in particular on an intact T-cell component. In present study, however, SLNT exhibited direct anti-tumor effects against HT-29 cells by suppressing cell proliferation and inducing cell apoptosis through ROS-mediated intrinsic and TNF-α-mediated extrinsic apoptotic pathways.

Apoptosis plays a crucial role in controlling tumorigenesis, and the incidence of apoptosis reportedly as colonocytes progress from normal epithelium to carcinoma [16, 17]. Up-regulation of Bcl-2, down-regulation of Bax, and p53 mutations have been suggested as possible candidates responsible for the change [18]. Therefore, induction of apoptosis is a promising strategy for the prevention and treatment of colon cancer [19]. In present study, SLNT suppressed HT-29 cells proliferation (Figure 1A) and significantly induced its apoptosis (Figure 1B–1D), indicating that SLNT exerted direct antitumor effect *in vitro*. Previous works revealed that Lentinan inhibited tumor growth in H22- and S180-bearing Balb/c mice through immunostimulation (greatly enhanced thymus index and spleen index) and the induction of apoptosis. In current study, SLNT (1.0 and 5.0 mg/kg) significantly suppressed tumor growth in athymic nude mice (Figure 2A–2C) with no changes in the spleen index (Table 1). This suggests the thymus may play key role in the immunoenhancement elicited by SLNT. At the same time, H&E staining showed that SLNT induced apoptosis within tumor tissues (Figure 2D), indicating direct antitumor activity that may have played a crucial role in this case.

Caspases, the central mediators of the apoptotic pathway, act as initiators or effectors [20]. Once activated, initiator caspases (caspase-2, -8, -9 and -10) cleave and activate downstream effector caspases (caspase-3, -6 and -7), which then cleave multiple intracellular proteins to induce apoptosis [21]. Caspase-3 is an essential effector caspase acting in both the intrinsic and extrinsic apoptosis pathways [22]. In this study, we demonstrated that SLNT activated caspase-3 in HT-29 cells and within tumors (Figure 3A–3C). The caspase-3 inhibitor Ac-DEVD-CHO significantly inhibited SLNT-induced apoptosis, though the effect was not complete (Figure 3E). This suggests SLNT-induced apoptosis depends to a large degree on activation of caspase-3, but caspase-3-independent pathways may also exist and require further investigation.

Apoptosis is triggered via intrinsic (mitochondrial) and extrinsic (death receptor) pathways [23]. The intrinsic pathway is activated by the loss of MMP and the release of cytochrome c from mitochondria into the cytosol. Once released, cytochrome c interacts with Apaf-1, ATP and procaspase-9 to form the apoptosome, which cleaves and activates caspase-9 and leads to activation of effector caspases [24–26]. Members of the anti-apoptotic (Bcl-2, Bcl-X_L_) and pro-apoptotic (Bax, Bad and Bid) Bcl-2 superfamily are intimately involved in the loss of MMP and release of cytochrome c [27], and the balance between these two groups is a critical regulator of this process [28, 29]. We found that SLNT treatment resulted in the loss of MMP (Figure 5) with significantly increased Bax and decreased Bcl-2 *in vitro* and *in vivo*, which led to the release of cytochrome c and activation of caspase-9 (Figure 4A–4D). Thus SLNT activated the intrinsic mitochondrial apoptosis pathway.

ROS arising from normal metabolism and xenobiotic exposure can be beneficial or harmful, depending on their concentration [30]. Overproduction of ROS is an upstream factor contributing to the activation of the mitochondrial apoptotic pathway [31, 32]. Here, we demonstrated that SLNT significantly increased ROS *in vitro* and *in vivo* (Figure 6A–6D), and the antioxidant NAC greatly inhibited SLNT-induced apoptosis (Figure 7A and 7B) and blocked up-regulation of cytosolic cytochrome c and Bax/Bcl-2 in cells (Figure 7C and 7D). This suggests ROS act as upstream mediators of SLNT-induced intrinsic apoptosis. However, NAC did not completely protect HT-29 cells from SLNT-induced apoptosis, suggesting the involvement of both ROS-dependent and -independent mechanisms. We therefore also considered the extrinsic apoptotic pathway.

The extrinsic apoptotic pathway is initiated by TNF-α binding to TNFR1 or FasL binding to Fas receptors. These associations lead to recruitment of adaptor molecules such as FADD and, in turn, activation of initiator caspase-8, and the caspase cascade, culminating in apoptosis [24]. Caspase-8 also can indirectly activate caspase-3 through cleavage of Bid to its truncated form (t-Bid) [33]. In present study, SLNT treatment led to ncreases in TNF-α levels and the activation of caspase-8, and thus the extrinsic apoptoic pathway, *in vitro* and *in vivo* (Figure 8). Unlike the rapid apoptosis induced by FasL, however, apoptosis is a late response to TNF-α [34]. TNF-α binding to TNFR-1 can also activate NF-κB, which inhibits of apoptosis through induction of anti-apoptotic factors [35]. Only when NF-κB activation is insufficient is apoptosis induced through caspase-8 activation. We found that SLNT effectively inhibited nuclear translocation of NF-κB in HT-29 cells and tumors (Figure 8A–8B and Figure 9).

**Figure 10:**
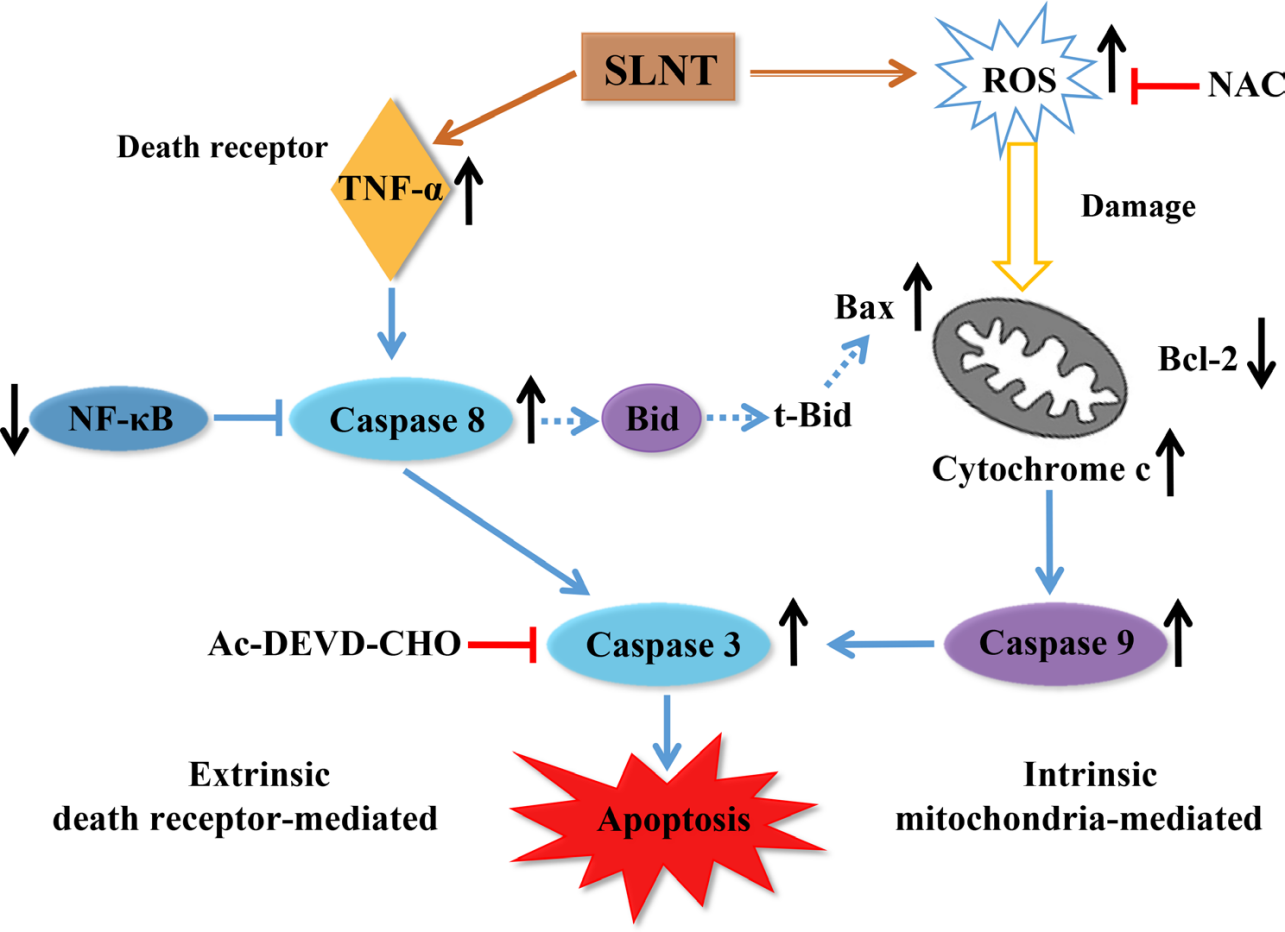
Flow diagram of the probable mechanism of direct antitumor effect of SLNT on HT-29 cells

In sum, our findings demonstrate that SLNT induces apoptosis through two pathways: (i) the intrinsic pathway mediated through overproduction of ROS, loss of MMP, increases in the Bax/Bcl-2 ratio and cytosolic cytochrome c, and activation caspase-9 and -3; (ii) the extrinsic pathway mediated through increases in TNF-α, inhibition of NF-κB, and activation of caspase-8 and -3 (Figure 10). Altogether, SLNT exerts direct antitumor effects on colon cancer cells both *in vitro* and *in vivo*. This suggests SLNT may be an effective natural agent for use in therapy against colon cancer.

## MATERIALS AND METHODS

### Reagents and antibodies

McCoy's 5A medium, fetal bovine serum (FBS), penicillin-streptomycin and trypsin-EDTA were obtained from Gibco (Grand Island, NY, USA). NAC, Ac-DEVD-CHO, DAPI and AO&EB, DCFH-DA, JC-1 stain kit, Cell and Tissue mitochondria isolation kits, Caspase-8 activity assay kit and BCA protein assay kit were obtained from Beyotime Biotech (Jiangsu, China). Nuclear-Cytosol kit was obtained from Applygen Technologies Inc (Beijing, China). Annexin V-FITC/PI assay kit, ROS and TNF-α ELISA kits were purchased from KeyGEN Biotech (Nanjing, China). Antibodies against Bax (^#^2772), Bcl-2 (^#^4223), Cytochrome c (^#^11940), Caspase-3 (^#^9662), cleaved caspase-3 (^#^9661), Caspase-8 (^#^4790), cleaved caspase-8 (^#^9496), Caspase-9 (^#^9508), NF-κB (p65, ^#^8242) and Histone H3 (^#^3638) were purchased from Cell Signalling Technology (Danvers, MA, USA). Anti-β-actin and anti-GAPDH antibodies, anti-mouse and anti-rabbit horseradish peroxidase (HRP)-conjugated secondary antibodies, 5-Fluorouracil (5-FU), MTT and standard sugar were purchased from Sigma (St. Louis, MO, USA). All other chemicals used were of analytical grade.

### Preparation and structure characterization of *Lentinus edodes* polysaccharide

The dried fruit bodies of *L. edodes* were obtained from FangXian (Hubei, China). The polysaccharide from *Lentinus edodes* (SLNT) was extracted, isolated and purified as previously described [11]. Briefly, after soaking with 95% alcohol, the fruit body residues were subjected to water extraction followed by ethanol precipitation, decoloration and ultrafiltration. A yield of around 1.91 g of SLNT was obtained from 250 g of *L. edodes*, which sugar content was approximately 98.14% and molecular weight was 623.5 kDa. UV spectrophotometer scanning showed that SLNT was absence of proteins and nucleic acids (data were not shown). The structure of SLNT was determined as previously described [11, 14]. GC-MS analysis revealed that the monosaccharide composition of SLNT was glucose alone. SLNT was further determined as polysaccharide containing β-D-glucans with pyranose by FT-IR analysis. Moreover, the types of glucosidic linkages and the molar ratios of them were analyzed by periodate oxidation and methylation. Results showed that β-(1→3)-D-glucose formed the backbone structure of SLNT and β-(1→6)-D-glucose formed side chains, which ratio was closer to 3:1. The basic structural unit of SLNT was shown in Supplementary Figure S1.

### Cell line and culture

Human colon cancer HT-29 cells, initially obtained from American Type Culture Collection (ATCC, Manassas, VA, USA), were maintained and grown in McCoy's 5A medium supplemented with 10% FBS and 1% penicillin/streptomycin. The cells were cultured at 37°C in a humidified atmosphere containing 5% CO_2._

### Cell viability assay

The HT-29 cells were cultured in the absence or presence of SLNT (0–800 μg/mL) or 5-FU (800 μg/mL) for 48 h and MTT assay was performed as previously described. Absorbance was measured at 570 nm using a microplate reader (Multiskan Mk3, Thermo Scientific, MA, USA).

### Flow cytometric analysis of apoptosis

HT-29 cells were grown in a 6-well plate at 2 × 10^5^ cells/well and treated with different concentrations of SLNT (400, 800, 1600 μg/mL) for 48 h, with untreated cells as negative control (NC). The cells were harvested, washed twice with PBS and re-suspended in 500 μL binding buffer. After being incubated with 5 μL of Annexin V-FITC and 5 μL of PI for 15 min, the cells were immediately analyzed by flow cytometry (BD, NJ, USA). Data analysis was performed using Flowjo V10 software.

### Morphological analysis

#### DAPI staining

HT-29 cells were seeded in a 24-well plate at 5 ×10^4^/well and treated with SLNT for 48 h. The cells were washed and stained with DAPI at room temperature for 15 min. Fluorescence was observed using a fluorescence microscope (DSY5000X, Chongqing, China).

### AO/EB staining

Dye mixture of 1 μL of AO (100 μg/mL) and 1 μL of EB (100 μg/mL) stained cells treated with SLNT or not for 10 min. After staining, cells were washed twice with PBS and visualized under fluorescence microscope (Chongqing, China).

### Detection of mitochondrial membrane potential (MMP)

The changes in the MMP of cells were assessed by JC-1 staining. Normally, healthy mitochondria was polarized and JC-1would be rapidly taken up into it as aggregates emitting in red. However, once ΔΨ_m_ depolarized, JC-1 would remain in the cytoplasm as monomers emitting in green. Thus the increase of ratio of green/red fluorescence indicated a loss of ΔΨ_m_. The detection was performed as manufacturer's instructions and analyzed by flow cytometry and fluorescence microscope.

### *In vivo* tumor xenograft study

All nude mice experiments were approved by the Institution Animal Care and Use Committee of Tongji Medical College, Huazhong University of Science and Technology (SYXK-2010-0057) and all mice were treated according to the guidelines set forth by the National Association for Assessment and Accreditation of Laboratory Animal Care. Thirty 5-week-old male athymic nude mice (BALB/c-nu) purchased from HFK Bioscience (Beijing, China) were maintained under specific pathogen-free conditions in the Central Animal Facility of Union Hospital (Wuhan, China), and acclimated before the experiment. HT-29 cells (3.75 × 10^6^) were injected subcutaneously into the right flank of nude mice (17–20 g in weight). Starting at 5 days post-injection, tumors were measured with calipers every other day and tumor size was calculated using the formula: V = (L × W^2^)/2, where L (mm) is the largest diameter and W (mm) is perpendicular to L. The treatments started at a day after injection of cells. Animals were randomly divided into five groups (*n* = 6/group) as follows: (i) sterile 0.9% saline (negative control); (ii) 0.2 mg/kg SLNT; (iii) 1 mg/kg SLNT; (iv) 5 mg/kg SLNT; and (v) 20 mg/kg 5-FU, and treated by intravenous (i.v.) injection through tail vein. SLNT was dissolved in sterile 0.9% saline and filtrated by 0.22 μm microporous membrane (Millipore, USA) prior to use. The doses of SLNT and 5-FU were selected on the basis of previous experiments [36, 37] and our preliminary studies. Injections were performed every other day for 21 days. In the end of treatments, mice were euthanized; tumor xenografts and spleens were excised, weighed and stored at −80°C. The antitumor activity of SLNT was expressed as an inhibition rate calculated as follows: inhibition rate (%) = [(Wa-Wb)/Wa] ×100%, where Wa and Wb were the average tumor weights of the control and experimental group, respectively.

### Measurement of ROS

*In vitro*, ROS levels were measured by DCFH-DA. Briefly, after treatment with SLNT for 48 h, HT-29 cells cultured in 6-well plates were incubated with DCFH-DA for 1 h at 37°C and then detected by fluorescence microscope and flow cytometry. *In vivo*, the concentration of ROS was measured by ELISA kits and analyzed at 450 nm by microplate reader.

### Measurement of TNF-α

The concentration of TNF-α *in vitro* and *in vivo* was measured by ELISA kits according to manufacturer's instructions. Briefly, the cell culture medium was harvested after SLNT treatment and the serum of mice was collected, separated and stored at −80°C before detection. The samples were added to ELISA plate with sample diluent (1:4) and incubated at 37°C for 30 min. Then, the plates were washed with wash buffer 5 times, added 50 μl HRP conjugate each plate except control, incubated at 37°C for 30 min and washed again. Next, substrate reagent (TMB) was added. The plates were incubated at 37°C for 15 min in the dark, followed by addition of 50 μl stop solution, and finally measured at 450 nm by microplate reader.

### H&E staining

Tumor tissues were fixed in 4% formalin, embedded in paraffin, sectioned and stained with haematoxylin and eosin (H&E) for histopathological assay. The image was captured at 400× by an optical microscope (Caikon XSP-11CD, Shanghai, China).

### Preparation of cytoplasmic and nuclear extracts

Cytoplasmic extracts of cells and tumors were isolated from mitochondria using the Cell and Tissue Mitochondria Isolation Kits, respectively, according to the manufacturer's instructions. Cells and tissues nuclear extracts were prepared using Nuclear-Cytosol Extraction Kit as previously described [38]. Cytoplasmic and Nuclear extracts were collected and stored at −80°C until use.

### Western blotting

To examine the expression of Bax, Bcl-2, cytosol Cytochrome c, cleaved caspase-3, cleaved caspase-8, caspase-9, nuclear NF-κB (p65), β-actin, GAPDH and Histone H3 proteins, Western blot analysis were performed as previously described [15]. The signal was visualized and evaluated by Image J software (National Institutes of Health, Bethesda, MD, USA).

### Immunofluorescence

Translocation of NF-κB (p65) from cytoplasm to nucleus was detected by immunofluorescence. Briefly, cells and thawed tumor sections were fixed and permeabilized. Then, the samples were washed with PBS, blocked with goat serum, probed with primary NF-κB p65 antibody for 1 h, and followed by incubation with cy3-conjugated secondary antibody for 1 h. Additionally, before observed using a fluorescence microscope at 400×, the samples were stained with DAPI for nuclear staining.

### Immunohistochemistry

HT-29 tumor samples were perfusion fixed with 4% paraformaldehyde and stored in 70% ethanol. Paraffin-embedded tumor sections (5 μm) were stained with antibodies against caspase-3 and cleaved caspase-8 using the method as previously described [15] and observed under an optical microscope at 400×.

### Caspase-8 activity assay

The activity of Caspase-8 in HT-29 cells was detected using Caspase-8 colorimetric activity assay kit, which is based on spectrophotometric detection of the chromophore *p*-nitroaniline (*p*NA) after cleavage from the labelled substrate Ac-IETD-*p*NA by the active enzyme. The caspase-8 activity was detected according to the manufacture's protocols. Absorbance (A) was measured at 405 nm using the microplate reader.

### Statistical analysis

Dates presented as mean ± SD and *p* values were determined using an unpaired Student's *t*-test from the GraphPad Prism version 5.0 software (San Diego, CA, USA). A *p*-value < 0.05 was considered as statistically significant.

## SUPPLEMENTARY MATERIALS



## Abbreviations

AO/EB: acridine orange and ethidium bromide
Ac-DEVD-CHO: N-Acetyl-Asp-Glu-Val-Asp-CHO
DAPI: 4, 6-diamidino-2-phenylindole
DCFH-DA: 2′, 7′-Dichlorofluorescein diacetate
5-FU: 5-Fluorouracil
NAC: N-acetylcysteine
NC: negative control
ROS: reactive oxygen species
SLNT: water-extracted polysaccharide from Lentinus edodes
TNF-α: tumor necrosis factor-α
