# Supplementary material for: A polysaccharide from Lentinus edodes inhibits human colon cancer cell proliferation and suppresses tumor growth in athymic nude mice
Source: Oncotarget. 2016 Nov 21;8(1):610–23. doi: 10.18632/oncotarget.13481 (PMC5352182; doi:10.18632/oncotarget.13481)
Supplement: Supplementary file 1 [file oncotarget-08-610-s001.pdf]

A polysaccharide from *Lentinus edodes* inhibits human colon cancer cell proliferation and suppresses tumor growth in athymic nude mice

Supplementary Materials

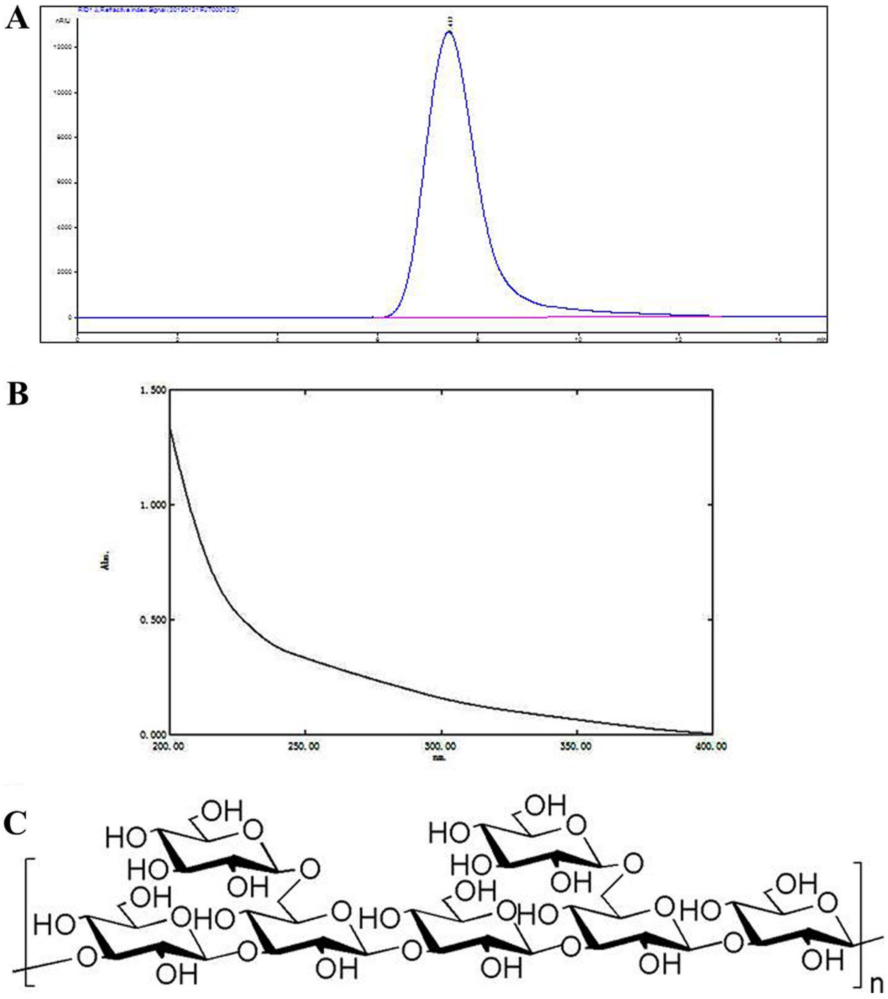

Supplementary Figure S1: The weight-average molecular weight of SLNT was 623.5 kDa, determined by HPGPC (A). No absorption at either 280 or 260 nm by UV spectrum, revealing the absence of protein and nucleic acid (**B**). The basic structural unit of SLNT (**C**).
